# Regulation of Adipocyte 11β-Hydroxysteroid Dehydrogenase Type 1 (11β-HSD1) by CCAAT/Enhancer-Binding Protein (C/EBP) β Isoforms, LIP and LAP

**DOI:** 10.1371/journal.pone.0037953

**Published:** 2012-05-25

**Authors:** Cristina L. Esteves, Val Kelly, Valérie Bégay, Tak Y. Man, Nicholas M. Morton, Achim Leutz, Jonathan R. Seckl, Karen E. Chapman

**Affiliations:** 1 Endocrinology Unit, University/BHF Centre for Cardiovascular Science, Queen’s Medical Research Institute, The University of Edinburgh, Edinburgh, United Kingdom; 2 Molecular Metabolism Group, University/BHF Centre for Cardiovascular Science, Queen’s Medical Research Institute, The University of Edinburgh, Edinburgh, United Kingdom; 3 Max Delbrüeck Center for Molecular Medicine, Berlin, Germany; Fudan University, China

## Abstract

11β-hydroxysteroid dehydrogenase type 1 (11β-HSD1) catalyses intracellular regeneration of active glucocorticoids, notably in liver and adipose tissue. 11β-HSD1 is increased selectively in adipose tissue in human obesity, a change implicated in the pathogenesis of metabolic syndrome. With high fat (HF)-feeding, adipose tissue 11β-HSD1 is down-regulated in mice, plausibly to counteract metabolic disease. Transcription of 11β-HSD1 is directly regulated by members of the CCAAT/enhancer binding protein (C/EBP) family. Here we show that while total C/EBPβ in adipose tissue is unaltered by HF diet, the ratio of the C/EBPβ isoforms liver-enriched inhibitor protein (LIP) and liver-enriched activator protein (LAP) (C/EBPβ-LIP:LAP) is increased in subcutaneous adipose. This may cause changes in 11β-HSD1 expression since genetically modified C/EBPβ^(+/L)^ mice, with increased C/EBPβ-LIP:LAP ratio, have decreased subcutaneous adipose 11β-HSD1 mRNA levels, whereas C/EBPβ^ΔuORF^ mice, with decreased C/EBPβ-LIP:LAP ratio, show increased subcutaneous adipose 11β-HSD1. C/EBPβ-LIP:LAP ratio is regulated by endoplasmic reticulum (ER) stress and mTOR signalling, both of which are altered in obesity. In 3T3-L1 adipocytes, 11β-HSD1 mRNA levels were down-regulated following induction of ER stress by tunicamycin but were up-regulated following inhibition of mTOR by rapamycin. These data point to a central role for C/EBPβ and its processing to LIP and LAP in transcriptional regulation of 11β-HSD1 in adipose tissue. Down-regulation of 11β-HSD1 by increased C/EBPβ-LIP:LAP in adipocytes may be part of a nutrient-sensing mechanism counteracting nutritional stress generated by HF diet.

## Introduction

11β-hydroxysteroid dehydrogenase type 1 (11β-HSD1) is highly expressed in liver and adipose tissue where it catalyses the regeneration of active glucocorticoids (corticosterone, cortisol) from inert 11keto- forms (11-dehydrocorticosterone, cortisone) thus increasing intracellular glucocorticoid action [1]. 11β-HSD1 expression is elevated selectively in adipose tissue of obese humans and in monogenic rodent genetic obesity, whereas levels in liver are unaffected or even decreased [2], [3], [4]. Transgenic over-expression of 11β-HSD1 in adipose tissue recapitulates the metabolic syndrome in mice, with visceral obesity, dyslipidemia, insulin resistance/diabetes and hypertension [2], [5]. In contrast, 11β-HSD1-deficiency or inhibition causes insulin-sensitization (including in humans), lowers fasting plasma glucose and lipid levels, reduces visceral adipose tissue mass and attenuates atherosclerosis [6], [7], [8]. Unexpectedly, high fat (HF) diet down-regulated 11β-HSD1 selectively in adipose tissue in mice and rats [9], [10], [11]. This down-regulation is greatest in obesity-resistant strains [9] suggesting it may be a mechanism to minimise metabolic disease with adiposity. Understanding the mechanisms of adipose-specific control of 11β-HSD1 is crucial to dissecting the pathogenesis of sensitivity/resistance to obesity.

Transcription of 11β-HSD1 is directly regulated by members of the CCAAT/enhancer binding protein (C/EBP) family of transcription factors in all tissues and cells studied [12], [13], [14], [15], [16]. The family comprises 6 members; C/EBPα, β, δ, γ, ε and ζ (or CHOP) [17]. C/EBPα, β, δ and CHOP are essential for adipocyte differentiation and function *in vitro* and *in vivo* [18], [19], [20], [21]. C/EBPα and β each occur as distinct isoforms arising from differential translation initiation or proteolysis [22], [23], [24]. C/EBPα produces 42 kDa (p42) and 30 kDa (p30) isoforms [22], [24], [25]. C/EBPβ exists as three major isoforms; the 38 kDa and 35 kDa liver-enriched activator protein isoforms (LAP* and LAP, respectively), which stimulate transcription, and the 20 kDa liver-enriched inhibitor protein (LIP) [24]. LIP lacks the transcription activation domain of C/EBPβ and is typically a dominant-negative regulator of C/EBP function [24]. The LIP:LAP ratio is thus an important determinant of C/EBPβ action [26], [27], [28].

*In vivo*, C/EBPα is the major known inducer of 11β-HSD1 transcription in liver, where C/EBPβ acts as a relative repressor [12]. In contrast, in adipose tissue, C/EBPβ is an activator [29]. Moreover, C/EBPβ is required for glucocorticoid-induction of 11β-HSD1 in A549 cells [16] as well as regulation by IL-1β, cAMP and ceramide in fibroblasts and 3T3-L1 preadipocytes [13], [14], [30]. However, the crucial transcriptional regulation of 11β-HSD1 in adipose tissue and mature adipocytes remains unexplored. Here, we have tested the hypothesis that C/EBPs are modulated by diet in mice and mediate the regulation of 11β-HSD1 in adipose.

## Materials and Methods

### Animals

C57BL/6J mice (Harlan UK Ltd., Oxon, UK) were housed in standard conditions on a 12 h light, 12 h dark cycle (lights on at 0700 h) at 21±1°C. Adult male mice (n = 16/group) were fed control diet (11% calories as fat; diet D12328, Research Diets, Inc., New Brunswick, NJ) or HF diet (58% calories as fat; diet D123331, Research Diets) for 6 weeks. Adult male C/EBPβ mutant mice, heterozygous C/EBPβ^(+/L)^ and homozygous C/EBPβ^ΔuORF^ and respective wild-type (WT) littermate control mice (n = 6–7/group) were generated as previously described [31], [32] and fed standard chow diet. All animal experiments were conducted in strict accord with accepted standards of humane animal care under the auspices of the Animal (Scientific Procedures) Act UK 1986 and following prior approval by the Home Office in UK or following prior approval by the Institutional Animal Care and Use Committee in Berlin, Germany.

### 3T3-L1 Adipocyte Differentiation and Transfection with siRNA

3T3-L1 murine preadipocytes (ATCC, American Type Culture Collection) were maintained and differentiated into mature adipocytes as previously described [33]. Briefly, 2d after reaching confluence, 3T3-L1 cells were induced to differentiate by the addition of 0.5 µM dexamethasone, 500 µM 3-isobutyl-1-methylxanthine and 5 µg/ml insulin for 2d. Thereafter, 3T3-L1 cell differentiation continued in medium supplemented with 5 µg/ml insulin alone. Experiments were performed 8–12d following induction of differentiation, unless otherwise stated. 3T3-L1 adipocytes were transfected with siRNA using DeliverX Plus (Panomics Inc., Fremont, CA) according to the manufacturer’s protocol. Fully differentiated adipocytes were gently detached by a brief wash with trypsin followed by collagenase type I (0.5 mg/ml; Invitrogen, Paisley, UK), and re-seeded at 1.5×10^5^ cells per well in a 12-well plate then transfected the next day with siRNA (32 pmol) in serum-free medium for 3 h followed by 21 h incubation in growth medium. All siRNAs were purchased from Applied Biosystems (Warrington, UK) and were scrambled (AM4611, negative control), C/EBPα (ID 101889), C/EBPβ (ID 288793) and CHOP (ID 288792). In the experiments designed to induce ER stress, 3T3-L1 adipocytes were treated with tunicamyin (4 µg/ml). Rapamycin (100 nM and 500 nM, 24 h) was used to inhibit the mammalian target of rapamycin (mTOR) in 3T3-L1 adipocytes.

### RNA Extraction and Analysis

Following dissection, subcutaneous adipose tissue (inguinal depot) was frozen on dry ice and stored at −70°C. Tissues were weighted on a balance model AA-160 (Denver Instrument Company, NY). RNA was extracted using Qiagen RNeasy Lipid Tissue Mini kit (Qiagen, West Sussex, UK). 3T3-L1 cells were harvested in Trizol. RNA (2 µg) was reverse transcribed using SuperScript III (Invitrogen) and quantified by real-time PCR on a LightCycler 480 (Roche) as described [16]. Primer-probe sets were purchased from Applied Biosystems: 11β-HSD1 (Mm00476182_m1), C/EBPα (Mm00514283_s1), C/EBPβ (Mm00843434_s1), C/EBPδ (Mm00786711_s1) and CHOP (Mm00492097_m1). Routinely, at least two internal standards were used from among 18S (Hs99999901_s1), β-actin (Mm00607939_s1) and TATA binding protein (TBP; Mm00446973_m1).

### Western Blot Analysis

Subcutaneous adipose tissue (inguinal depot) was homogenized in lysis buffer (0.125M Tris-HCL, pH 6.8, 2% sodium dodecyl sulphate, and 10% glycerol) in the presence of a protease inhibitor cocktail (P2714; Sigma-Aldrich, Dorset, UK) and heated at 100°C. 3T3-L1 adipocytes were harvested directly into lysis buffer and processed similarly. Electrophoresis was carried out on 4–12% NuPage Bis-Tris gels (Invitrogen). After transfer, blots were probed with antibodies to C/EBPα, -β, -δ (1∶500 dilution of stock 200 µg/ml; Santa Cruz Biotechnology Inc., Santa Cruz, CA), CHOP (1∶1000 dilution; ABcam, Cambridge, UK) and β-tubulin (1∶10000 dilution; Chemicon/Millipore, Watford, UK) and secondary antibodies anti-rabbit IgG-HRP (1∶2000 dilution from stock 400 µg/ml Santa Cruz Biotechnology Inc.), anti-mouse IgG-HRP (1∶4000 dilution; Cell signaling, Danvers, MA), anti-rabbit IgG Alexa Fluor 700 (Invitrogen) and anti-mouse IgG (Rockland Immunochemicals Inc. Gilbertsville, PA). The resulting bands were analyzed by ImageJ (NIH, USA) or the Odyssey Infrared Imaging System (LI-COR Biosciences Ltd, Cambridge, UK), as appropriate.

**Figure 1:**
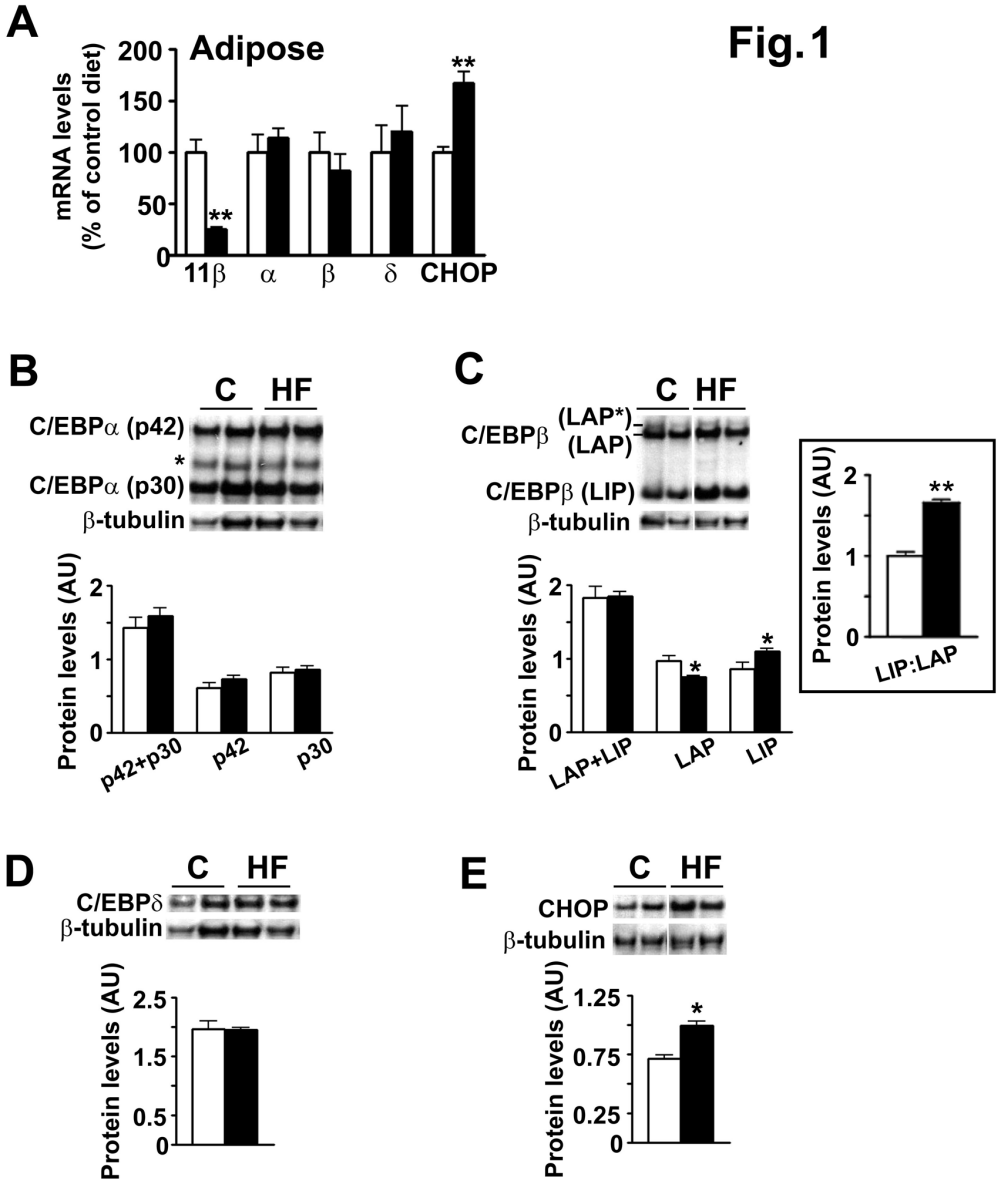
Expression of 11β-HSD1, C/EBPα, β, δ and CHOP in adipose tissue of HF diet-fed mice compared to control diet-fed mice. (A) Real-time PCR measurements of levels of mRNA encoding 11β-HSD1, C/EBPα, β, δ, and CHOP in adipose tissue of HF-fed (black bars) and control diet-fed (white bars) mice. Levels of mRNA, normalized to TBP, are expressed relative to levels in control diet-fed mice, arbitrarily set to 100%. (B-E) Representative western blots (50 µg protein/lane) and quantification of blots probed with; (B) C/EBPα antibody showing levels of the 42 kDa (p42) and 30 kDa (p30) isoforms of C/EBPα as well as total C/EBPα (p42+p30) levels (the intermediate immunoreactive protein indicated by * is likely to be a proteolytic product of p42); (C) C/EBPβ antibody showing levels of LAP (38 kDa LAP* +35 kDa LAP) and the 20 kDa LIP isoforms of C/EBPβ as well as total C/EBPβ (LAP+LIP) levels (inset shows LIP:LAP ratio); (D) C/EBPδ antibody and (E) CHOP antibody showing levels of respective proteins in adipose tissue of mice fed HF diet (HF, black bars) or control diet (C, white bars). Blots were stripped and reprobed with β-tubulin antibody, as loading control. In C/EBPβ and CHOP westerns, all samples were analysed in the same gel but not all in adjacent lanes. C/EBP levels were quantified relative to β-tubulin levels using ImageJ analysis and are expressed in arbitrary units (AU). All values are mean±SEM; n = 16/group (A) or 4/group (B-E). *, p≤0.05; **, p<0.001.

### Chromatin Immunoprecipitation (Chip) Assays

ChIP assays were carried out using an Upstate EZ ChIP kit (Millipore, Billerica, MA) according to the manufacturer’s protocol. Briefly, differentiating 3T3-L1 cells (8 h after induction of differentiation) or fully mature adipocytes (differentiated for 9d) in 15 cm tissue culture dishes were incubated with dimethyl adipimidate.2 HCL (10 mM in PBS; Thermo Fisher Scientific Cramlington, UK) for 30 min in the dark followed by 1% (v/v) formaldehyde. Cells were lysed in sodium dodecyl sulphate buffer (provided in the kit). Chromatin was sheared by sonication using a Soniprep 150 (MSE; Beckenham, Kent, UK) with eight 10 s pulses, at maximum amplitude, keeping cells on ice between pulses. Immunoprecipitations were carried out with 5 µg C/EBP or control rabbit IgG antibody (Santa Cruz). After reversal of cross-links, purified DNA was amplified by standard PCR (35 cycles of 94°C 30 s, 56°C 1 min, 72°C 1 min) or real-time PCR (95°C 5 s, 65°C 1 min) as above, using primers spanning the following C/EBP-binding sites in the 11β-HSD1 promoter [12]: footprint FP1 and FP2 (5′-CTAGTGCTGCCTGAGACTACTCC-3′ and 5′-TTTTCATAACTGCCATCAAACAG-3′), FP3 and FP4 (5′- CAGTAGGAGATGCTCAGGAACC-3′ and 5′-AGAGCAACGATTGGCTTTGG-3′), FP9 (5′-ACTTGCCTGAGAGTCTTGAACTG-3′ and 5′-AGCAAAATAAGCTCAAGGGAAAA-3′) and a C/EBPβ binding site in the C/EBPα promoter (5′-GGTGACTTAGAGGCTTAAAGGAG-3′ and 5′-GTGACTTTCCAAGGCGGTGAGT-3′). Primers spanning an 11β-HSD1 intronic sequence not predicted to bind C/EBP were used as a control (5′-TCAAACATCCAGGTTCTTTCATT-3′ and 5′-CATACCACATAAACCACACATGG-3′). Data are expressed relative to levels of input DNA in immunoprecipitations.

**Figure 2:**
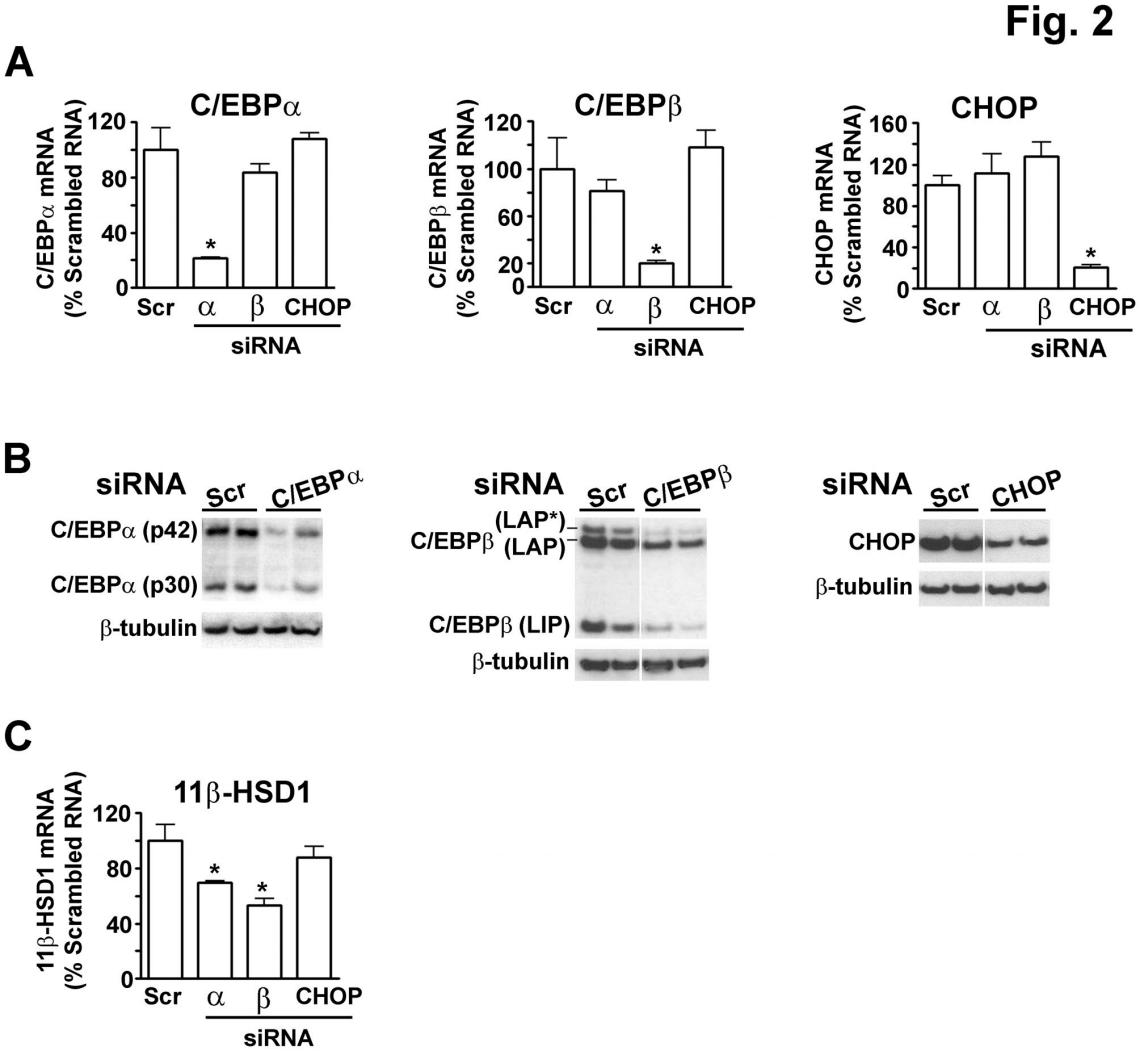
Transfection of fully differentiated 3T3-L1 adipocytes with C/EBPα or C/EBPβ siRNA decreases 11β-HSD1 mRNA levels, whilst CHOP siRNA has no effect. (**A**) Real-time PCR measurement of levels of mRNA encoding C/EBPα (left panel), C/EBPβ (centre panel) and CHOP (right panel), 24 h after transfection of fully differentiated 3T3-L1 adipocytes with scrambled RNA (Scr; as control) or siRNAs (32 pmol) targeting C/EBPα, β or CHOP. Data are normalized to TBP and expressed relative to levels in cells transfected with scrambled RNA (arbitrarily set to 100%). Values are mean±SEM of 3 different experiments (independent adipocyte differentiations) with each siRNA treatment tested in triplicate. *, Significantly different from scrambled RNA, p≤0.05. (**B**) Representative western blots showing levels of the 42 kDa (p42) and 30 kDa (p30) isoforms of C/EBPα (left panel; 20 µg protein/lane), 38 kDa LAP*, 35 kDa LAP and 20 kDa LIP isoforms of C/EBPβ (centre panel; 40 µg protein/lane) and CHOP (right panel; 20 µg protein/lane), 24 h after transfection of 3T3-L1 adipocytes with scrambled RNA or respective siRNAs targeting C/EBPα, β or CHOP. Blots were stripped and reprobed with β-tubulin antibody, as loading control. In C/EBPβ and CHOP westerns all samples were analysed in the same gel but not all in adjacent lanes. (**C**) Real-time PCR measurement of mRNA encoding 11β-HSD1, 24 h after transfection of 3T3-L1 mature adipocytes with scrambled RNA or siRNA (32 pmol) targeting C/EBPα, β or CHOP. Data are normalized to TBP and expressed relative to levels in cells transfected with scrambled RNA (arbitrarily set to 100%). Values are mean±SEM of 3 to 4 different experiments (independent adipocyte differentiations) with each siRNA treatment tested in triplicate. *, Significantly different from scrambled RNA, p≤0.05.

### Statistical Analysis

All data were analyzed by Student’s *t* test or ANOVA followed by *post hoc* Tukey, Fisher LSD or Dunnet tests using SigmaStat 2.03 statistical software. Significance was set at p≤0.05.

**Figure 3:**
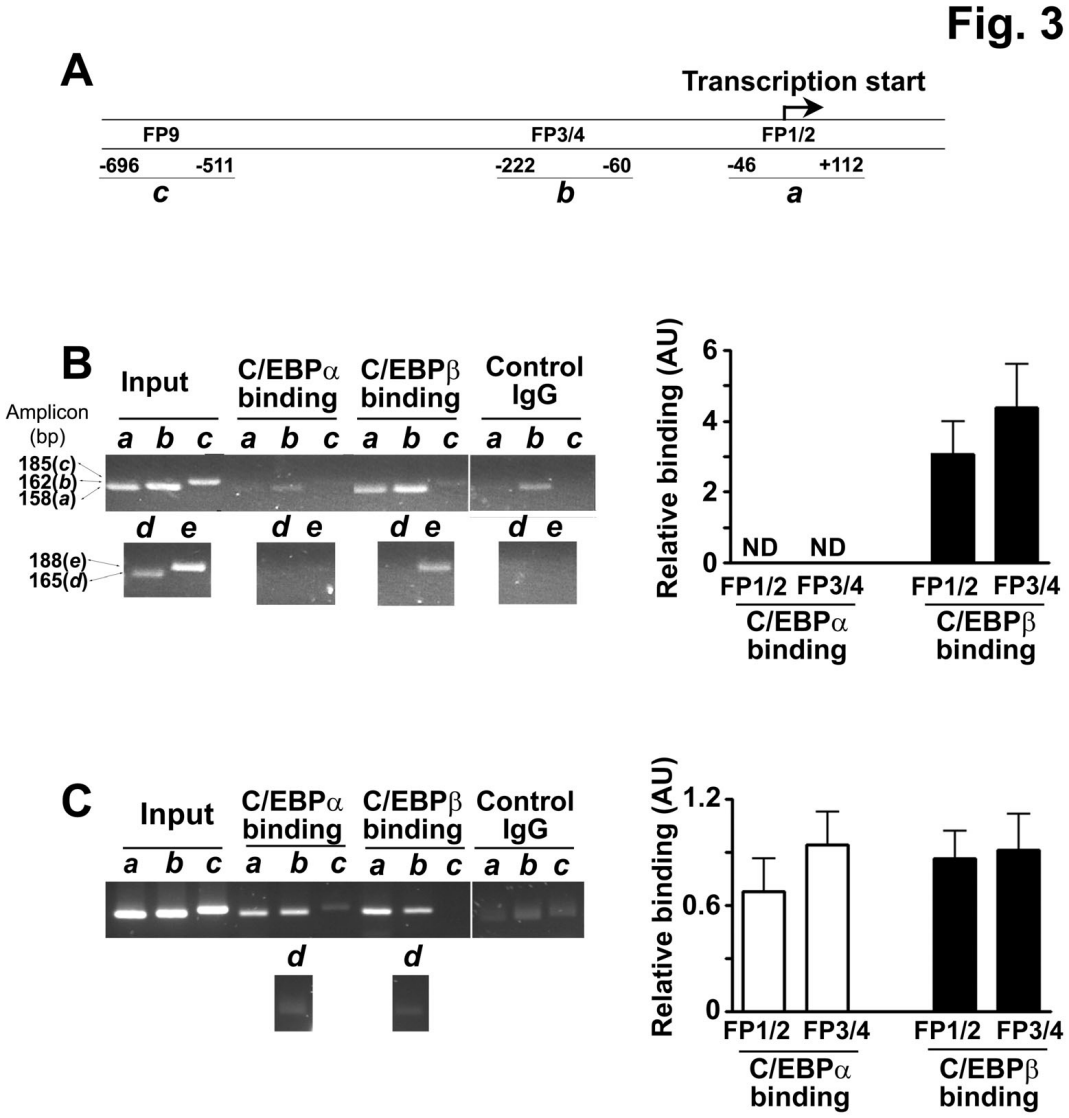
Binding of C/EBPα and C/EBPβ to the promoter of 11β-HSD1 throughout 3T3-L1 adipocyte differentiation. (**A**) Relevant C/EBP binding sites in the mouse 11β-HSD1 promoter; FP1 to 4 and 9. The start of transcription (+1) [12] is indicated with a bent arrow. The indicated C/EBP binding sites are highly conserved between the rat and mouse 11β-HSD1 promoters [12], [13] and are named according to the sites in the rat promoter [12]. PCR products amplified in the ChIP experiment are indicated (*a* to *c*). (**B**) and (**C**) ChIP assays carried out to examine C/EBP binding to the 11β-HSD1 promoter in (**B**) differentiating 3T3-L1 adipocytes (8 h after induction of differentiation) and (**C**) fully differentiated adipocytes (9d after induction of differentiation). Left panels show representative gels of ChIP assays following immunoprecipitation with C/EBPα or C/EBPβ antibody. Control reactions contained rabbit IgG. PCR reactions (35 cycles) were carried out on input DNA (input) or on immunoprecipitated DNA with primers spanning FP1/2 of the 11β-HSD1 promoter (lanes marked *a*), FP3/4 (lanes marked *b*), FP9 (lanes marked *c*), an intronic sequence from the 11β-HSD1 gene not predicted to bind C/EBP (lanes marked *d*) and a C/EBPβ binding site in the C/EBPα promoter [44] (lanes marked *e*). In (**B**), all PCR products were visualized in the same gel but not in adjacent lanes, whereas in (**C**), all PCR reactions were performed at the same time but were visualized in different gels. Right panels show real-time PCR quantification of C/EBPα and C/EBPβ binding to the FP1/2 and FP3/4 regions of the 11β-HSD1 promoter. Reactions were performed with the same primers used for standard PCR as detailed in Methods. ND, not detected. Values are mean±SEM from 2 to 3 independent experiments and each sample was analysed in triplicate.

## Results

### Effect of HF Diet on 11β-HSD1 and C/EBP Expression in Mouse Adipose Tissue

Mice fed HF diet for 6 weeks were heavier (HF, 30.8±0.52 *vs* control diet, 26.3±0.34 g; p<0.01) with increased subcutaneous adipose tissue weight (HF, 0.022±0.001 *vs* control, 0.012±0.001 (w/w) corrected for body weight; p<0.01), while liver weight was unchanged (HF, 0.049±0.001 *vs* control, 0.046±0.003 (w/w) corrected for body weight). 11β-HSD1 mRNA was down-regulated in adipose tissue by HF diet both in subcutaneous (Fig. 1A) and visceral (mesenteric) depots (data not shown), but was unchanged in liver (data not shown), consistent with previous data showing down-regulation of 11β-HSD1 mRNA and enzyme activity in adipose tissue of mice fed HF diet [9], [11]. To test whether altered C/EBP expression may underlie the dietary regulation of 11β-HSD1, we examined C/EBPα, β, δ and CHOP expression in adipose tissue. HF diet did not change C/EBPα, β or δ mRNA levels, while CHOP mRNA levels were increased in subcutaneous adipose tissue (Fig. 1A) and mesenteric [11] adipose tissue (data not shown). Because 11β-HSD1 and C/EBP mRNA levels showed the same pattern of changes in both adipose depots of HF-fed (vs control) animals, subcutaneous adipose, which is more abundant, was used for subsequent analyses. Consistent with mRNA levels, western blot analysis showed an increase in CHOP protein levels but no alteration in total C/EBPα (p42+ p30 isoforms), total C/EBPβ (LAP* + LAP + LIP) or C/EBPδ (Fig. 1B–E) protein levels with HF diet. However, HF diet reduced adipose tissue levels of the C/EBPβ-LAP*+LAP isoforms, concomitantly increasing levels of C/EBPβ-LIP (Fig. 1C), resulting in a significant increase in the C/EBPβ-LIP:LAP ratio (Fig. 1C, inset).

**Figure 4:**
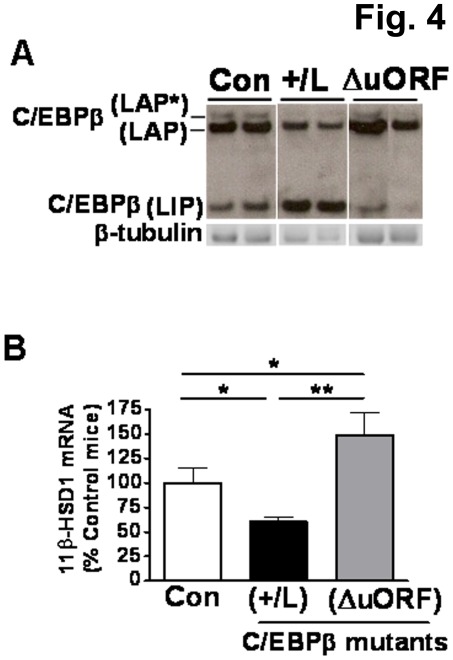
Altered C/EBPβ-LIP:LAP ratio *in vivo* affects 11β-HSD1 expression in adipose tissue. (**A**) Representative western blot (40 µg protein/lane) showing levels of C/EBPβ-LAP (38 kDa LAP* +35 kDa LAP) and -LIP (20 kDa) isoforms in the adipose tissue of C/EBPβ mutants C/EBPβ^(+/L)^ (+/L), C/EBPβ^ΔuORF^ (ΔuORF) and control (Con) mice. Blots were stripped and reprobed with β-tubulin antibody, as loading control. All samples were analysed in the same gel but not all in adjacent lanes. (**B**) Real-time PCR measurement levels of mRNA encoding 11β-HSD1 in adipose tissue of wild type control mice (Con, white bar), C/EBPβ^(+/L)^ (+/L, black bar) and C/EBPβ^ΔuORF^ (ΔuORF, grey bar). C/EBPβ^(+/L)^ mice are heterozygous for an allele of C/EBPβ in which the normal gene has been replaced by C/EBPβ-LIP (a “knock-in”) [31] and C/EBPβ^ΔuORF^ is homozygous for the deletion of upstream ORF codon [32]. Adipose 11β-HSD1 mRNA levels, normalized to TBP, are expressed relative to levels in control mice (arbitrarily set to 100%) and are mean±SEM; n = 6–9/group. *, p≤0.05; **, p<0.001.

**Figure 5:**
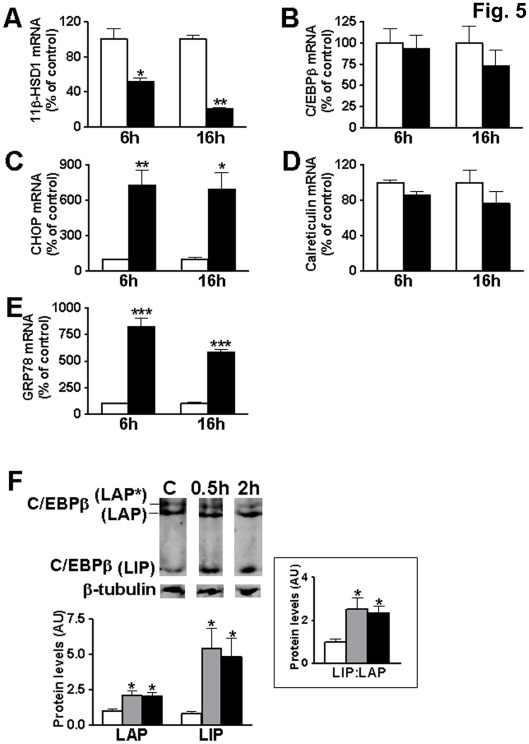
Tunicamycin-induced ER stress reduces 11β-HSD1 expression in 3T3-L1 adipocytes. (**A–E**) Real-time PCR measurement of levels of mRNA encoding (**A**) 11β-HSD1, (**B**) C/EBPβ, (**C**) CHOP, (**D**) calreticulin and (**E**) GRP78 in untreated differentiated 3T3-L1 adipocytes (white bars) or treated with tunicamycin (4 µg/ml, black bars) for 6 or 16 h. Data are normalized to TBP and expressed relative to levels of control cells (arbitrarily set to 100%). (**F**) Representative western blot (40 µg protein/lane) and quantification showing levels of C/EBPβ-LAP (38 kDa LAP* +35 kDa LAP) and -LIP (20 kDa) isoforms. Inset shows C/EBPβ-LIP:LAP ratio in untreated 3T3-L1 adipocytes (C, white bar) or treated with tunicamycin for 0.5 h (0.5 h, grey bar) or 2 h (2 h, black bar). Blots were stripped and reprobed with β-tubulin antibody, as loading control. All samples were analysed in the same gel but not in adjacent lanes. C/EBPβ levels were quantified relative to β-tubulin and are expressed in arbitrary units (AU). Values are mean±SEM of 2–3 independent adipocyte differentiations with each treatment tested in duplicate or triplicate. *, Significantly different from control. *, p≤0.05; **, p<0.001; ***, p<0.0001.

### C/EBPα and C/EBPβ, but not CHOP, Regulate Expression of 11β-HSD1 in 3T3-L1 Adipocytes

Increased CHOP levels or increased C/EBPβ-LIP:LAP ratio could plausibly reduce adipose 11β-HSD1 expression. The effect of CHOP on 11β-HSD1 expression has not been reported. Moreover, although C/EBPβ regulates 11β-HSD1 expression during preadipocyte differentiation [13], [29], any role in mature adipocytes has not been tested. To investigate the requirement for C/EBPα, C/EBPβ and CHOP in 11β-HSD1 expression in adipocytes, 3T3-L1 preadipocytes were fully differentiated into mature adipocytes, which show high endogenous expression of 11β-HSD1 [33], and then transfected with siRNAs to decrease levels of C/EBPα, C/EBPβ or CHOP. Measurement of mRNA and protein levels confirmed the reduction in expression of appropriate C/EBPs with siRNA, notably reducing all isoforms of C/EBPα and C/EBPβ (Fig. 2A, B) but increasing C/EBPβ-LIP:LAP ratio (scrambled RNA, 0.5±0.03 *vs* C/EBPβ siRNA 0.7±0.02). The results also showed that reduced expression of one of the C/EBPs in mature 3T3-L1 adipocytes did not affect mRNA levels of the other C/EBPs tested (Fig. 2A). Both C/EBPα and C/EBPβ siRNA reduced 11β-HSD1 mRNA levels in 3T3-L1 adipocytes (Fig. 2C), demonstrating that both these members of the C/EBP family play a key role in maintaining adipocyte expression of 11β-HSD1. In contrast, although levels of CHOP were reduced in 3T3-L1 adipocytes by siRNA (Fig. 2A, B), this did not alter 11β-HSD1 mRNA levels (Fig. 2C).

**Figure 6:**
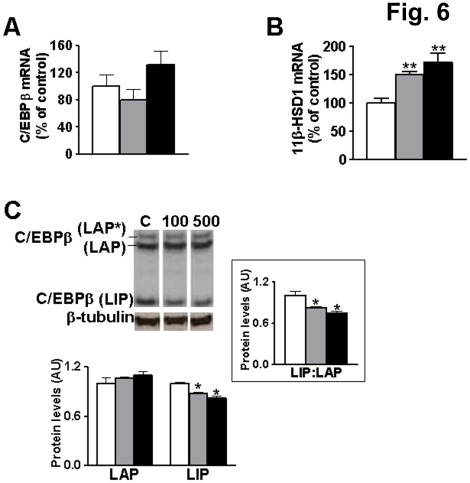
Inhibition of mTOR in 3T3-L1 adipocytes reduces C/EBPβ-LIP:LAP ratio and increases 11β-HSD1 expression. Real-time PCR measurement of levels of mRNA encoding (**A**) C/EBPβ and (**B**) 11β-HSD1 in 3T3-L1 adipocytes (white bars) or following treatment with 100 nM (grey bars) or 500 nM (black bars) rapamycin for 24 h. Data are normalized to TBP and expressed relative to levels of control cells (arbitrarily set to 100%). (**C**) Representative western blot (40 µg protein/lane) and quantification showing levels of C/EBPβ-LAP (38 kDa LAP* +35 kDa LAP) and -LIP (20 kDa) isoforms and inset showing C/EBPβ-LIP:LAP ratio in untreated 3T3-L1 adipocytes (C, white bars) or following treatment with 100 nM (100, grey bars) or 500 nM (500, black bars) rapamycin for 24 h. Blots were stripped and reprobed with β-tubulin antibody, as loading control. All samples were analysed in the same gel but not in adjacent lanes. C/EBPβ levels were quantified relative to β-tubulin and are expressed in arbitrary units (AU). Values are mean±SEM of up to 3 independent adipocyte differentiations, each treatment tested in triplicate. *, Significantly different from control. *, p≤0.05; **, p<0.001.

### C/EBPβ Locates to the Promoter of 11β-HSD1 throughout Adipocyte Differentiation Whilst C/EBPα Binds Only in Differentiated Adipocytes

Previous reports showed C/EBPβ binding to the 11β-HSD1 promoter in undifferentiated 3T3-L1 preadipocytes [13], [14], but this has not been described in adipocytes. To determine if C/EBPs interact with the 11β-HSD1 promoter in immature and fully mature adipocytes, binding of C/EBPα and β was assayed by chromatin immunoprecipitation (ChIP). The proximal promoter of the rat 11β-HSD1 gene contains 4 C/EBP binding sites between -196 and +44; footprints (FP) 1 to 4, which are conserved in the mouse promoter [12], [13] (Fig. 3A). C/EBPβ was bound to the proximal 11β-HSD1 promoter early during adipocyte differentiation (when 11β-HSD1 expression is low) (Fig. 3B) as well as in fully differentiated 3T3-L1 adipocytes (Fig. 3C). In contrast, C/EBPα was only bound to the 11β-HSD1 proximal promoter in fully differentiated 3T3-L1 adipocytes, which highly express 11β-HSD1 [33]. Neither C/EBPα nor C/EBPβ were detected at FP9 in the promoter of 11β-HSD1 (also conserved between rat and mouse) (Fig. 3B, C). Thus C/EBPβ interacts directly with the 11β-HSD1 promoter in mature adipocytes.

### Increased C/EBPβ-LIP:LAP Ratio *in vivo* Decreases 11β-HSD1 mRNA Levels in Adipose Tissue, an Effect Reversed in Mice with Decreased C/EBPβ-LIP:LAP Ratio

To test whether altered C/EBPβ-LIP:LAP ratio can similarly alter adipose 11β-HSD1 expression *in vivo*, we measured 11β-HSD1 mRNA levels in subcutaneous adipose tissue of C/EBPβ^(+/L)^ and C/EBPβ^ΔuORF^ mice which have increased and decreased C/EBPβ-LIP:LAP ratio, respectively. In C/EBPβ^(+/L)^ mice, a “knock-in” of C/EBPβ-LIP (L allele) replaces the normal C/EBPβ gene [31], increasing C/EBPβ-LIP:LAP ratio (Fig. 4A). In C/EBPβ^ΔuORF^ mice, deletion of the upstream open reading frame (ΔuORF allele) prevents translation of C/EBPβ-LIP [32], thus decreasing C/EBPβ-LIP:LAP ratio (Fig. 4A). 11β-HSD1 mRNA levels were significantly lower in adipose tissue of C/EBPβ^(+/L)^ mice (increased C/EBPβ-LIP:LAP ratio) than in wild-type controls (Fig. 4B). The reverse was observed in C/EBPβ^ΔuORF^ mice (Fig. 4B), demonstrating that, *in vivo*, the C/EBPβ-LIP:LAP ratio controls the transcription of 11β-HSD1.

### 11β-HSD1 mRNA Levels are Down-regulated by ER Stress in 3T3-L1 Adipocytes

Elevated C/EBPβ-LIP:LAP ratio and CHOP levels, observed in adipose tissue of HF-fed mice, may be a result of ER stress [34], [35]. Treatment of differentiated 3T3-L1 adipocytes with tunicamycin, an inducer of ER stress [35], significantly reduced 11β-HSD1 mRNA levels at 6 h, with a more pronounced effect at 16 h (Fig. 5A). Similar results were obtained with an alternative ER stress-inducer, thapsigargin (data not shown). Down-regulation of 11β-HSD1 mRNA was accompanied by up-regulation of genes increased by ER stress; CHOP and the 78 kDa glucose-regulated protein (GRP78) (Fig. 5C, E), though calreticulin mRNA levels were unaffected at this stage (Fig. 5D). C/EBPβ mRNA levels were not altered (Fig. 5B) whilst, as expected, C/EBPβ-LIP:LAP ratio was increased, and both C/EBPβ-LAP and -LIP were up-regulated (Fig. 5F). These results show that induction of ER stress, which increases the C/EBPβ-LIP:LAP ratio, also down-regulates 11β-HSD1 in 3T3-L1 adipocytes.

### mTOR Regulates the Expression of 11β-HSD1 in 3T3-L1 Adipocytes

The mTOR pathway, which is responsive to nutrient availability and growth factors, controls many aspects of cellular metabolism and regulates the production of C/EBPβ-LIP and -LAP isoforms [24]. To test the importance of this pathway for the transcriptional regulation of 11β-HSD1, rapamycin was used to inhibit mTOR in differentiated 3T3-L1 adipocytes. Rapamycin did not alter C/EBPβ mRNA levels (Fig. 6A) or C/EBPβ-LAP, but decreased C/EBPβ-LIP, resulting in a lower C/EBPβ-LIP:LAP ratio (Fig. 6C). This was associated with increased 11β-HSD1 mRNA levels (Fig. 6B). These results show that the mTOR signalling pathway regulates 11β-HSD1 transcription in adipocytes, plausibly via modulation of the C/EBPβ-LIP:LAP ratio.

## Discussion

This study emphasises the key role played by members of the C/EBP family in the regulation of 11β-HSD1 transcription in adipocytes. However, the main finding here is that the striking down-regulation of 11β-HSD1 in adipose tissue of mice in response to HF feeding is likely due to changes in post-transcriptional processing of C/EBPβ generating an increased C/EBPβ-LIP:LAP ratio. Indeed, knock-down of C/EBPβ in fully differentiated mature 3T3-L1 adipocytes showed that C/EBPβ is required for normal 11β-HSD1 expression in differentiated adipocytes, consistent with a previous report of reduced 11β-HSD1 expression in adipose tissue of C/EBPβ-deficient mice [29]. In differentiating 3T3-L1 adipocytes, C/EBPβ binds to the C/EBPα promoter and induces its expression [20]. Importantly, in contrast, knock-down of C/EBPβ in fully differentiated adipocytes did not affect levels of C/EBPα, suggesting a direct role for C/EBPβ in adipocyte 11β-HSD1 expression, independent of its effect on C/EBPα expression. Indeed, C/EBPα mRNA and protein levels were unchanged in adipose tissue of HF diet-fed mice. However, it cannot be excluded that C/EBPβ may also exert its effect by regulating C/EBPα transactivation potential. Nevertheless, supporting a direct role, C/EBPβ was bound to the 11β-HSD1 promoter in mature adipocytes as well as in 3T3-L1 preadipocytes during the differentiation process. C/EBPα, in contrast, was only bound to the 11β-HSD1 promoter in differentiated adipocytes, as expected from its later appearance during differentiation [20]. Thus, both C/EBPα and C/EBPβ play a direct and positive role in regulation of 11β-HSD1 transcription in differentiated adipocytes, but C/EBPβ alone is crucial in differentiating cells. This is likely to impact on the 11β-HSD1 mRNA levels in these cells, where C/EBPα is absent or in low levels. In adipose tissue, C/EBPβ and CHOP were the only C/EBPs changed by HF diet. Although CHOP generally represses transcription [36], it can also be a co-activator [37]. However, siRNA-mediated knock-down of CHOP showed that it plays no role in 11β-HSD1 expression in fully differentiated 3T3-L1 adipocytes, although we cannot rule out that increased CHOP levels may contribute to the down-regulation of 11β-HSD1 in adipose tissue with HF diet.

An altered C/EBPβ-LIP:LAP ratio, like the HF-induced increase in CHOP, might be a response to ER stress as a consequence of a high demand for ER activity with nutrient excess [34]. The C/EBPβ-LIP:LAP ratio is controlled by the double stranded RNA-dependent protein kinase (PKR, a target of ER stress [38]) and mTOR signalling pathways through the eukaryotic translation factors eIF-2α and eIF-4E, respectively [24]. Indeed, diet-induced obesity in mice results in the activation of ER stress pathways in metabolically relevant tissues, such as adipose and liver, with increased phosphorylation of the PKR-like kinase and its substrate eIF-2α, which are key indicators of ER stress [34]. mTOR may contribute to the ER stress response as well [39]. Our results show that both induction of ER stress and inhibition of mTOR signalling alter C/EBPβ-LIP:LAP ratio and modulate 11β-HSD1 mRNA levels in 3T3-L1 adipocytes. ER stress and mTOR signalling pathways are sensitive to cellular nutrients and energy homeostasis, suggesting that the increased C/EBPβ-LIP:LAP ratio in obese adipose tissue may result from activation of these nutrient/stress sensing mechanisms leading to the down-regulation of 11β-HSD1.

Alterations in C/EBPβ-LIP:LAP ratio are likely to impact upon other metabolically-important genes, many of which, like 11β-HSD1, are regulated by C/EBPβ [40], [41]. Indeed, the gene encoding phosphoenolpyruvate carboxykinase, a C/EBPβ-regulated enzyme with key roles in hepatic gluconeogenesis and adipose glycerogenesis, is down-regulated by an increased LIP:LAP ratio [26]. C/EBPβ-null mice have decreased gluconeogenesis and lipolysis during fasting or diabetes [40], [41], resist diet-induced obesity [42] and exhibit attenuated ER stress and inflammatory responses [43]. Importantly, mice deficient in C/EBPβ lack both LIP and LAP isoforms and so are not informative regarding the relevance of the C/EBPβ-LIP:LAP ratio. In contrast, gene targeted C/EBPβ^(+/L)^ and C/EBPβ^ΔuORF^ mice, have altered levels of the C/EBPβ isoforms. C/EBPβ^ΔuORF^ mice show activation of acute-phase response genes after partial hepatectomy, suggesting that C/EBPβ-LIP may be a natural restraint of inflammation [32]. However, the impact of the C/EBPβ-LIP:LAP ratio on 11β-HSD1 expression has not been previously reported. Here, we demonstrate that manipulation of the C/EBPβ-LIP:LAP ratio regulates the expression of 11β-HSD1, in agreement with the modulation of 11β-HSD1 mRNA levels observed in mouse adipose tissue by diet.
